# Treatment of multiple traumatized anterior teeth associated with an alveolar bone fracture in a 20-year-old patient: A 3-year follow up

**DOI:** 10.4317/jced.51374

**Published:** 2014-10-01

**Authors:** Vicente Faus-Matoses, María Martínez-Viñarta, Teresa Alegre-Domingo, Ignacio Faus-Matoses, Vicente J. Faus-Llácer

**Affiliations:** 1 DDS, MSc. Master of Restorative Dentristy and Endodontics, Department of Stomatology, Medicine and Dental School, Valencia University, Spain; 2 DDS, MSc. Master in Prosthetic Dentistry, Department of Stomatology, Medicine and Dental School, Valencia University, Spain; 3 MD, DDS, MSc, PhD. Master of Restorative Dentristy and Endodontics, Department of Stomatology, Medicine and Dental School, Valencia University, Spain

## Abstract

Intrusive luxation is a type of recognizable luxation injury represented by a deeper axial displacement of the tooth toward the alveolar bone. Treatment strategies include waiting for the tooth to return to its position, immediate surgical repositioning, and repositioning through dental traction by orthodontic devices. The aim of this case report was to present the management of severe dental trauma and later restoration following IADT. A 20-year-old patient was presented after fainting at home four hours before, resulting in a dento-alveolar trauma. Clinical examinations revealed a traumatic intrusion, in 1.2, 1.1 and 2.1, uncomplicated crown fractures in 1.1 and 2.1 and a complicated crown-root fracture in 2.2. The diagnosis was confirmed with CBCT. Following IADT protocol, the emergency treatment consisted of the surgical repositioning and semi-rigid splinting using orthodontic wire-composite, replacing the buccal bone plate, and postoperative instructions to the patient regarding oral hygiene. After 2 weeks the root canal treated and filled with fiberglass posts in 1.2, 1.1, 2.1 and 2.2. Splint was removed after 4 weeks and the IADT reassessment protocol followed, with revisions at 6-8 weeks, 6 months, a year and annual reviews for 5 years. A year after the treatment, the traumatized teeth were restored with minimally invasive preparations of feldspathic ceramic. Esthetics and function were recorded with a 3-year follow-up period.

** Key words:**Intrusive luxation, dental trauma, crown-root fracture, dento-alveolar trauma, permanent tooth, CBCT.

## Introduction

Intrusive luxation is a type of recognizable luxation injury characterized by a deeper axial displacement of the tooth toward the alveolar bone (1), representing 0.3-1.9% of dental trauma in permanent teeth (2). The highest incidence occurs in boys between 6-12 years old (3) which makes treatment of these injuries difficult (4). The upper central incisors are commonly the most affected teeth [93.3%]. Predominant etiologic factors are falls at home [60%], followed by bicycle injuries (2,5).

Severe dental traumas can involve numerous injuries, including intrusion [33.5%], an associated crown fracture intrusion [60.5%], or a combination of intrusion and coronal or root fractures [6%] (2). In most cases it affects only one tooth [46.3%], followed by two teeth [32.4%] and three or more teeth [21.3%] (2). Most of the intruded teeth are displaced 2-8 mm into the alveolar bone (2), with infrequent intrusions occurring over 6 mm (5).

Three types of treatment have been proposed for traumatic intrusions in permanent teeth: watchful waiting with passive repositioning [41.7%], immediate surgical repositioning [58.3%] (5) or orthodontic traction. The indications for treatment strategy depend on the stage of root development, the severity of the intrusion, the presence of alveolar fracture or the number of teeth involved in multiple intrusions. The focus must be on the elimination or attenuation of the injury to avoid future complications (4,6-8).

The aim of this case report was to present the management of severe dental trauma in a 20-year-old man and later restoration following the protocol of International Association for Dental Traumatology [IADT].

## Case Report

A 20-year-old male was referred to the Department of Stomatology, Master of Restorative Dentistry and Endodontics, Medicine and Dental School, Valencia University, Spain, after fainting at home four hours before, resulting in a dento-alveolar trauma. His medical history included absence seizures as a child and the use of an orthodontic device for eight months before the trauma.

**Figure 1:**
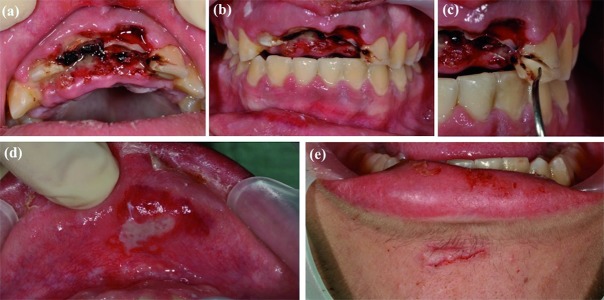
(a) Oclusal view, traumatic intrusion in 1.2, 1.1 and 2.1. (b) Vestibular view, traumatic intrusion in 1.2, 1.1 and 2.1. (c) Complicated crown-root fracture in 2.2. (d) Lower lip laceration. (e) Chin lateration.

**Figure 2:**
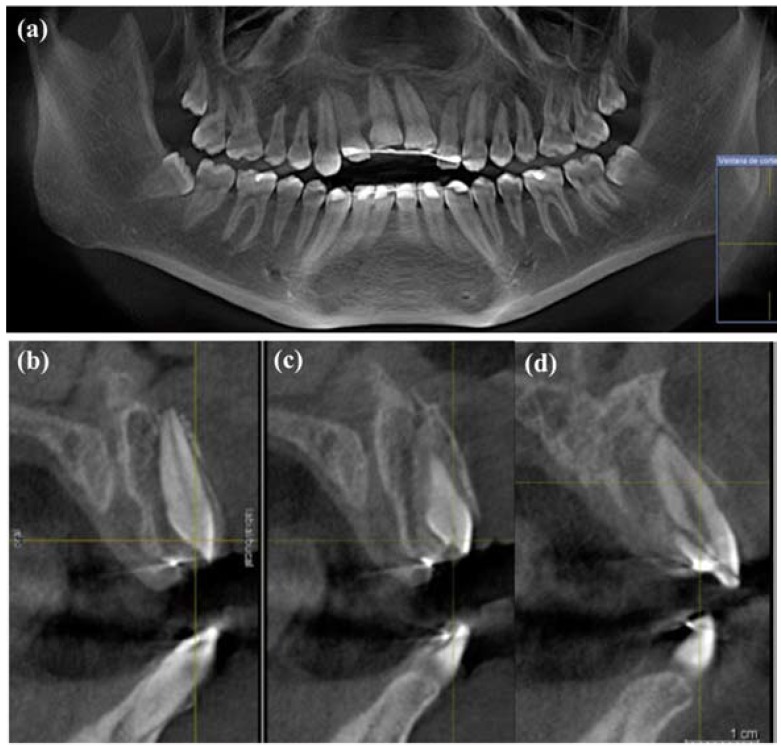
(a) The initial orthopantomography. (b) CBCT radiographic examination, showed an alveolar bone fracture and traumatic luxation in 1.1. (c) CBCT radiographic examination showed a fracture of the vestibular cortical bone. (d) CBCT radiographic examination showed a complicated crown-root fracture in 2.2.

Intraoral examination revealed a traumatic intrusion, greater than 7 mm, in 1.2, 1.1 and 2.1, uncomplicated crown fractures in 1.1 and 2.1 and a complicated crown-root fracture in 2.2 (Fig. 1). The patient also suffered lower lip and chin laceration (Fig. 1). The diagnosis was confirmed with CBCT radiographic examination, which showed a fracture of the vestibular cortical bone (Fig. 2).

**Figure 3:**
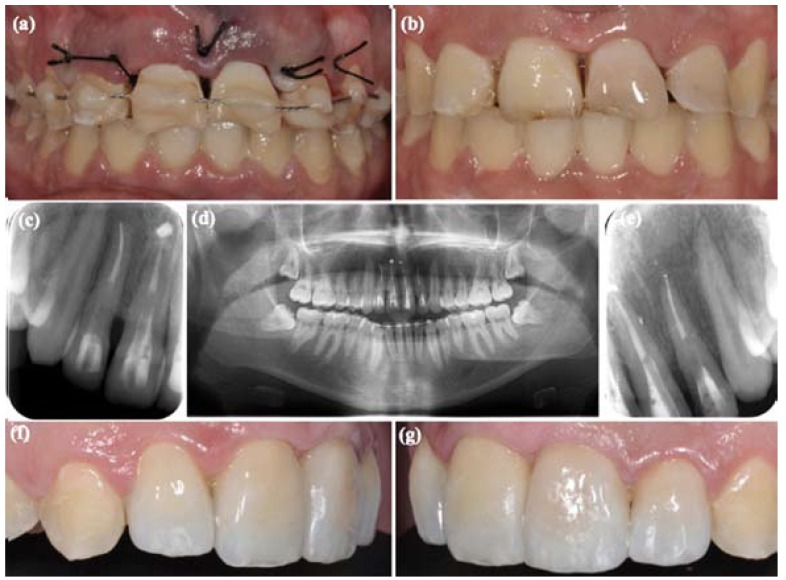
(a) Surgical repositioning and semi-rigid splinting using orthodontic wire-composite. (b) Splint was removed. (c) Root canal treatment and filling with glass fiber posts in 1.1 and 1.2. (d) Orthopantomography, 2 weeks follow-up. (e) Root canal treatment and filling with fiberglass posts in 2.1 and 2.2. (f) Lateral left view, the traumatized teeth were restored with minimally invasive preparations of feldspathic ceramic. (g) Lateral right view.

Following IADT protocol, the emergency treatment consisted, under local anesthesia [epinephrine 1:100,000], on the surgical repositioning, removal the fragment of 2.2 with forceps and semi-rigid splinting using orthodontic wire-composite (Fig. 3); replacing the buccal bone plate, and postoperative instructions to the patient regarding oral hygiene using chlorhexidine 0.1% rinses for 2 weeks. No antibiotic coverage was administered, because there were no significant differences in healing if antibiotics are used or not (6).

After 2 weeks the stitches were removed and the root canal treated was performed in 1.2, 1.1, 2.1 and 2.2 using rotary instruments [ProTaper Universal, Dentsply Maillefer, Ballaigues, Switzerland] and sealed with thermoplasticized gutta-percha [Thermafil, Dentsply Maillefer, Ballaigues, Switzerland]. The teeth were then restored with fiberglass posts [Normopost, Normon, Biolonen, Saronno, Italy] (Fig. 3). In addition, in 2.2 a palatal gingivectomy and ostectomy was performed to prevent the invasion of the periodontal space of the future restoration. Splint was removed after 4 weeks (Fig. 3) and the IADT reassessment protocol followed with revisions at 6-8 weeks, 6 months, a year and annual reviews for 5 years (9). A year after the treatment, the traumatized teeth were restored with minimally invasive preparations of feldspathic ceramic [Creation Willi Geller International GmbH, Meiningen, Austria] (Fig. 3).

## Discussion

Dentoalveolar trauma involving multiple teeth are rare (10), so its treatment remains a challenge for professionals. This study aims to report a multiple trauma with severe intrusions of three teeth, uncomplicated crown fractures, complicated crown-root fracture, and vestibular cortical bone fracture.

Intrusive dislocations are diagnosed based on clinical signs and symptoms, such as lack of occlusal alignment, bleeding and a dry thud sound when tapped (4). In this study, radiographic examination revealed that the periodontal ligament space had disappeared as had the height difference between the apexes of the traumatized teeth and their counterparts.

The incorporation of CBCT has significantly improved the ability to accurately diagnose traumatic injuries (11). The use in this study of CBCT images of the vestibular cortical bone provided high diagnostic accuracy that resulted in a good treatment strategy.

Three types of treatments for intruded teeth are proposed without reaching a consensus for an optimal alternative: passive spontaneous reposition, surgical reposition and orthodontic reposition (5). The treatment strategy will depend on the development stage of the root apex, the severity of the intrusion, the presence of alveolar fractures or multiple intrusions, and should be focused on the elimination or attenuation of post-injury complications (2,4,6-8).

The correct treatment for intruded teeth with complete root formation and intrusion greater than 7 mm is, according to Diangelis *et al*. (9), a surgical extrusion and stabilizing the tooth using a flexible splint for 4-8 weeks. However, Andresen *et al*. (6) conducted a study of 140 intruded teeth and evaluated their healing according to the type of splinting and found no significant differences. In this study, a semi-rigid wire-composite splint was used as suggested by Berthold *et al*. (12), since it is also suitable for cortical bone fractures. To prevent pulp necrosis, root canal treatment of the teeth intruded is recommended after 2-3 weeks of the surgical extrusion (6).

The emergency treatment for complicated crown-root fractures, according to Diangelis *et al*. (9), is the temporary stabilization of the lost fragment with the adjacent tooth. Subsequently, the fragment should be removed, the root canals treated and coronal restoration made with fiberglass post. This procedure must be preceded by a gingivectomy and sometimes by an osteoplasty and ostectomy. This type of treatment is only suitable for crown-root fractures with subgingival palatal extension, as in this case report in which there was a complicated crown-root fracture of 2.2 with subgingival palatal extension.

Successful healing following injury depends mainly on the development stage of the root. There is less risk of complications in cases of incomplete root formation. This is probably because of a softer bone surrounding the teeth, which requires smaller penetrations for periodontal ligament injuries. Crown fractures [with exposed dentin] had a negative influence on pulp healing. The extent of the intrusion was partly influenced by the degree of intrusion being higher than 7 mm. Finally, the adjacent intruded teeth had a negative influence on the healing of the marginal bone between the intruded teeth (4,13).

In conclusion, severe dental trauma involving three or more teeth are a challenge for dentists. The teeth involved in the trauma should be regularly monitored in order to detect and treat possible complications arising as a result. After the three years control period, it was concluded that following the IADT protocol proved successful in preserving the dental health, and satisfying both the aesthetic and functional wishes of the patient. Understanding current treatment guidelines will improve the care of dental trauma in general and emergency care, in particular.
